# Psychobiosocial States as Mediators of the Effects of Basic Psychological Need Satisfaction on Burnout Symptoms in Youth Sport

**DOI:** 10.3390/ijerph17124447

**Published:** 2020-06-21

**Authors:** Milena Morano, Laura Bortoli, Montse C. Ruiz, Claudio Robazza

**Affiliations:** 1 Parisi-De Sanctis Institute, MIUR (Italian Ministry of Education, University and Research), 71121 Foggia, Italy; milenamorano@gmail.com; 2 School of Medicine and Health Sciences, “G. d’Annunzio” University of Chieti-Pescara, 66013 Chieti, Italy; 3 BIND-Behavioral Imaging and Neural Dynamics Center, Department of Medicine and Aging Sciences, “G. d’Annunzio” University of Chieti-Pescara, 66013 Chieti, Italy; c.robazza@unich.it; 4 Faculty of Sport and Health Sciences, University of Jyväskylä, 40014 Jyväskylä, Finland; montse.ruiz@jyu.fi

**Keywords:** youth sport participation, motivation, Self-Determination Theory, emotions, IZOF model

## Abstract

Sport participation in youngsters has been associated with long-lasting psychosocial and health-related benefits as well as increased levels of physical exercise in adulthood. The objective of this study was to examine some psychological factors of fundamental importance in enhancing sport participation and preventing burnout. A sample of 520 girls and boys aged 13–18 years, practicing individual or team sports, took part in a cross-sectional study to assess basic psychological need satisfaction, psychobiosocial states, and burnout symptoms. The specific purpose was to examine the mediation effects of emotion-related (i.e., functional/dysfunctional) psychobiosocial states on the relationship between basic psychological need satisfaction (i.e., autonomy-choice, competence, and relatedness) and burnout symptoms (i.e., emotional/physical exhaustion, a reduced sense of accomplishment, and sport devaluation). Competence need satisfaction was found to be the most influential variable, with direct and indirect effects on burnout components, in particular, on a reduced sense of sport accomplishment. Overall, findings support the usefulness of investigating psychobiosocial states in youth sport and indicate that functional psychobiosocial states, as consequences of environmental motivational aspects, can have a significant effect on contrasting burnout symptoms.

## 1. Introduction

Sport participation in youngsters results in psychosocial and health-related benefits, fostering youth development. A large body of research evidence highlights that organized youth sport participation is associated with increased levels of exercise in adulthood [1,2], and positive psychological outcomes and skills, such as increased self-esteem, emotional control, academic achievement, leadership, and social skills [3,4]. According to Côté and Fraser-Thomas [5], the sporting experience has the potential to promote the attainment of three important objectives, as it provides opportunities to be physically active, learn personal and social life skills, and improve sport-specific skills and performance. Although the focus in sport is on physical and technical development, performance cannot be the only primary goal [6]. Many research studies reflect the growing interest in those psychosocial factors that enhance the individual motivation underlying sport involvement and adherence [7,8]. Despite the increasing research attention, however, several areas surrounding motivation in sport warrant further investigation for both research and applied purposes.

In particular, the study of the relationships between motivational and emotional factors in youth sport can provide important indications for enhancing the sport experience and preventing burnout in youngsters [9]. As a source of pleasant emotions for young athletes, sport experience has been examined in light of a number of theoretical frameworks. A prominent holistic approach to the study of emotional experiences in the sport context is the Individual Zones of Optimal Functioning (IZOF) model [10,11] (for reviews, see [12,13,14]). The IZOF model focuses on a variety of psychobiosocial states related to performance. These states are defined as situational, multimodal, and dynamic manifestations of total human functioning, whereby emotion is viewed in a multidimensional manner and considered a fundamental component of an individual’s experience [11]. Psychobiosocial states include at least eight emotional and non-emotional interactive modalities (i.e., affective, cognitive, motivational, volitional, bodily-somatic, motor-behavioral, operational, and communicative) that describe athletes’ perceptions of personal and environmental conditions. These states are contended to exert a functional or dysfunctional impact on performance and, more generally, on sport experience. As conceptualized within the IZOF model, psychobiosocial states have been studied in youth sport as related to contextual and individual motivational aspects [15,16,17,18]. Findings have supported the feasibility and utility of adopting the IZOF model in combination with other theoretical frameworks, including Achievement Goal Theory [19,20].

The Basic Psychological Needs Theory (BPNT) [21,22,23] has been one of the most relevant contemporary theoretical approaches to the study of motivational aspects in youth sport. Grounded on the broad framework of the Self-Determination Theory, the BPNT highlights the role of social and environmental support, and assumes that the satisfaction of the three basic psychological needs of competence, autonomy, and relatedness underpins self-determined motivation [24,25]. According to the BPNT, competence reflects the perception of being able to effectively interact with the environment and to experience a sense of mastery or achievement, autonomy refers to experience choice and freedom in action, and relatedness is defined as the feeling of being connected to and accepted by significant others in a specific context. The satisfaction of these three basic psychological needs is crucial for optimal development and well-being [25].

Most studies based on the BPNT have been focused on the positive outcomes derived from basic psychological need satisfaction. Adie et al. [21] extensively examined the relationships between basic need satisfaction and indices of both well- and ill-being. Perceptions of high energy and vitality were considered as a signal of positive physical and psychological functioning, whereas perceptions of physical and emotional exhaustion were employed as an indicator of ill-being. Emotional/physical exhaustion is one of the three central dimensions of the burnout construct. The other two are a reduced sense of accomplishment, or the perception of inability to reach personal goals, and sport devaluation, defined as a loss of personal interest in sport participation [26]. Even though youth sport participation generally offers many psychosocial benefits, an increased pressure to begin high-intensity training at young ages, with an excessive focus on competition and performance, can lead to burnout feelings [27,28,29], which can eventually prompt young athletes to drop out of their sport.

### Study Purpose

Based on the literature mentioned above, the objective of the current study was to examine the relationship between the perceived satisfaction of basic psychological needs, psychobiosocial states, and burnout symptoms in young athletes. Psychological need satisfaction, as a situational antecedent of burnout, was expected to exert protective effects against burnout symptoms, and therefore, a negative relationship between the two variables was predicted. Psychobiosocial states were hypothesized to mediate this relationship. Specifically, functional states were expected to be positively related to psychological need satisfaction and negatively related to burnout symptoms. On the other hand, dysfunctional states were expected to manifest an opposite relationship, that is, a negative relationship with need satisfaction and a positive one with burnout symptoms.

## 2. Materials and Methods

### 2.1. Sample

A convenience sample of 520 young athletes took part in our cross-sectional study. Participants were recruited from several sport clubs located in Central Italy. After the exclusion of 13 outliers identified through data analysis, the final sample (*n* = 507) was divided into two age categories of 13–15-year-olds (*n* = 276, 116 girls and 160 boys; *M* age = 13.93, *SD* = 0.81) and 16–18-year-olds (*n* = 231, 104 girls and 127 boys; *M* age = 17.17, *SD* = 0.78) practicing individual sports (girls: gymnastics *n* = 26, track and field *n* = 25, rhythmic gymnastics *n* = 20, tennis *n* = 12, swimming *n* = 10, fencing *n* = 7, skiing *n* = 2; boys: tennis *n* = 60, fencing *n* = 9, track and field *n* = 6, swimming *n* = 6, skiing *n* = 2) or team sports (girls: volleyball *n* = 52, basketball *n* = 25, soccer *n* = 24, futsal *n* = 13, handball *n* = 4; boys: soccer *n* = 86, basketball *n* = 72, rugby *n* = 23, futsal *n* = 13, handball *n* = 10). The competition level in the whole sample was national (*n* = 90), regional (*n* = 261), or local (*n* = 156). The mean years of sport participation was 5.41 (*SD* = 2.73) for the 13–15-year-olds and 6.73 (*SD* = 3.72) for the 16–18-year-olds. The participants were usually engaged for a minimum of three training sessions per week, each of 2 h.

### 2.2. Measures

#### 2.2.1. Psychological Needs Satisfaction

The Basic Needs Satisfaction in Sport Scale (BNSSS) [30] is a measure of psychological need satisfaction in sport, consisting of 20 items loaded into five factors: Competence, Autonomy-choice, Internal perceived locus of causality, Volition, and Relatedness.

In the current study, we used 14 items of the BNSSS to assess Competence (5 items; e.g., “I am skilled at my sport”), Autonomy-choice (4 items; e.g., “In my sport, I get opportunities to make decisions”), and Relatedness (5 items; e.g., “In my sport, there are people who I can trust”). We used these three subscales in the assessment of participants because the focus of the study was on the three basic psychological needs of competence, autonomy, and relatedness that characterize self-determined motivation [24,25].

The three subscales from the BNSSS were adapted to the Italian language using back-translation procedures [31]. Responses were rated on a 7-point Likert scale ranging from 1 (*not true at all*) to 7 (*very true*). Previous research results [30] supported the factor structure of the scale and showed acceptable internal consistency (alpha coefficients) of the subscale scores of Competence (0.77), Autonomy-choice (0.85), and Relatedness (0.77). The factor structure of the instrument has also been supported in a sample of Spanish athletes [32]. The composite reliability indices were 0.88, 0.95, and 0.95 for the Competence, Autonomy-choice, and Relatedness subscales, respectively.

#### 2.2.2. Psychobiosocial States

The psychobiosocial states scale, trait version (PBS-ST) [33], was derived from the English version of the Individualized Profiling of Psychobiosocial States [34] and adapted to the Italian language. The PBS-ST scale comprises 15 items, 8 functional and 7 dysfunctional, to assess seven modalities of a performance-related state (i.e., affective, cognitive, motivational, volitional, bodily-somatic, motor-behavioral, and operational). Each item includes 3 or 4 descriptors of a similar experience exerting a functional (+) or dysfunctional (−) impact on performance. The affective modality comprises three rows of synonym adjectives assessing functional pleasant states (+), “enthusiastic, confident, carefree, joyful”; functional anger (+), “fighting spirit, fierce, aggressive”; and dysfunctional anxiety (−), “worried, apprehensive, concerned, troubled”. In the other six modalities, two rows of adjectives measure functional or dysfunctional states. For example, the functional cognitive (+) modality includes the “alert, focused, attentive” adjectives, while the dysfunctional cognitive (−) modality encompasses “distracted, overloaded, doubtful, confused”; the functional motor-behavioral (+) modality comprises “relaxed-, coordinated-, powerful-, effortless-movement”, while the dysfunctional motor-behavioral (−) modality includes “sluggish, clumsy, uncoordinated, powerless-movement”. The participants in this study were asked to rate the intensity of the psychobiosocial items on a 5-point Likert scale, ranging from 0 (*not at all*) to 4 (*very, very much*), referring to how they usually feel in their sport context. In a sample of male and female athletes from different sports, the two-factor (i.e., functional and dysfunctional) solution was supported, with chi-square/degrees of freedom (*χ*^2^/df) = 1.478, comparative fit index (CFI) = 0.950, Tucker–Lewis index (TLI) = 0.942, root-mean-square error of approximation (RMSEA) (90% CI) = 0.038 (0.023−0.051), and standardized root-mean square residual (SRMR) = 0.048.

#### 2.2.3. Burnout

The 15-item Athlete Burnout Questionnaire (ABQ) [26] consists of three scales with 5 items each to measure emotional/physical exhaustion (e.g., “I feel overly tired from my sport participation”), a reduced sense of accomplishment (e.g., “I am not performing up to my ability in sport”), and sport devaluation (e.g., “I’m not into sport like I used to be”). The participants were asked to indicate how often they felt a certain way during the current season on a 5-point Likert scale ranging from 1 (*almost never*) to 5 (*almost always*). The Cronbach’s alpha values for the subscales ranged from 0.84 to 0.88 [35]. In a sample of Italian adolescent athletes, the alpha values were 0.81, 0.72, and 0.77 for the emotional/physical exhaustion, reduced sense of accomplishment, and sport devaluation subscales, respectively [9].

### 2.3. Procedure

The study was conducted after approval from the local ethics committee (n. 1813/09coet) and was in compliance with the ethical standards outlined in the Declaration of Helsinki. First, we contacted sport managers and coaches to explain the general purpose of the investigation and to obtain authorization to approach the athletes. After permission was granted, athletes were assessed within training facilities, in quiet locations, before regular practice sessions during the competitive season. The multi-section questionnaire was administered in groups of up to five participants. Participants were given an explanation of the general purpose of the study, presented with instructions indicating that there were no right or wrong answers, and informed that individual responses would remain confidential. They also received instructions designed to minimize social desirability biases. Written informed consent was obtained from participants over 18 and from the parents of those under 18 years of age.

### 2.4. Data Analysis

Data were initially examined for missing values, potential univariate or multivariate outliers, and violations of assumptions of normality, linearity, multicollinearity, and homoscedasticity [36]. Confirmatory factor analysis (CFA) was then conducted on the data of the whole sample to examine the factorial validity of the three measures (i.e., BNSSS three scales, PBS-ST, and ABQ). CFA was accomplished with *Mplus* version 8.4 [37] using the maximum likelihood (MLM) estimator to identify maximum likelihood parameter estimates with standard errors and a mean-adjusted chi square (*χ*^2^) test statistic, which are robust to non-normality. Regarding the sample size for model estimation, we adopted the more restrictive indications of Hair et al. [36], who suggest a minimum sample size of 500, based on the complexity of the model and the basic characteristics of the measurement model—namely, a model with a large number of constructs and some constructs with lower communalities. Consistent with commonly accepted indications for model-fit criteria [38,39], acceptable fit was inferred with values of *χ*^2^/df lower than 5, a CFI and TLI close to 0.95, a RMSEA from 0.05 to 0.08, and an SRMR smaller than 0.05 [36,40,41].

Descriptive statistics, Pearson product-moment correlation coefficients, Cronbach’s alpha (α) values, composite reliability (CR) values, omega (ω) coefficients [42], and the average variance extracted were computed for all the studied variables, namely, Competence, Autonomy-choice, Relatedness, Functional psychobiosocial states, Dysfunctional psychobiosocial states, Emotional/physical exhaustion, Reduced sense of accomplishment, and Sport devaluation. The magnitudes of the correlation coefficients were interpreted according to Zhu’s [43] indications, namely, 0–0.19 = no correlation, 0.20−0.39 = low correlation, 0.40−0.59 = moderate correlation, 0.60−0.79 = moderately high correlation, and > 0.80 = high correlation. Multivariate analysis of variance (MANOVA) was performed on the mean scores of the dependent variables to examine differences by gender and age categories (i.e., 13–15 vs. 16–18).

Finally, path analysis was performed to test the hypothesized relationships between basic psychological need satisfaction, psychobiosocial states, and burnout in the data of the four gender-by-age categories. To examine whether Functional or Dysfunctional psychobiosocial states would mediate the relationship between basic need satisfaction and burnout variables, we used the Hayes’ [44] PROCESS macro for SPSS (SPSS Inc., Chicago, IL, USA). Along with computation of standard regression statistics, this tool enables the estimation of path coefficients, standard errors, effect size indices, and bias-corrected bootstrap confidence intervals for the indirect effects. Bootstrap 95% confidence intervals not including the value zero suggest that the indirect effects are significant at *p* < 0.05.

## 3. Results

The data screening of the whole sample showed no missing data. Thirteen outliers (seven in the younger sample and six in the older sample) were identified using Mahalanobis’ distance criterion. After removal, the final sample consisted of 507 participants.

The CFA results for the whole sample (*N* = 507) are reported in Table 1. The analysis yielded unsatisfactory fit indices for the BNSSS (Competence, Autonomy-choice, and Relatedness latent variables) and acceptable ones for the PBS-ST (Functional and Dysfunctional latent variables). On both measures, the examination of the modification indices suggested to correlate two error terms of indicators of each factor. After model re-specification, the fit of the two measures was improved, thus supporting the three-factor structure of the BNSSS and the two-factor structure of the PBS-ST. CFA of the ABQ (Emotional/physical exhaustion, Reduced sense of accomplishment, and Sport devaluation latent variables) also yielded poor fit indices. Scale scrutiny showed two items with low factor loadings (< 0.40) on the Sport devaluation subscale. After item removal, the fit improved. All the factor loadings of the three measures were > 0.40. Acceptable reliability indices (α, CR, and ω) and average variance extracted were also found. They are presented in Table 2 together with descriptive statistics and correlation coefficients for the data of the whole sample. After having established the factorial validity and reliability of the instruments, the mean scores of the latent factors of measures were then used for MANOVA and path analyses.

MANOVA yielded significant results by gender—Pillai’s trace = 0.119, *F*(8, 496) = 8.397, *p* < 0.001, and *η*_p_^2^ = 0.119—and by age category—Pillai’s trace = 0.063, *F*(8, 496) = 4.203, *p* < 0.001, and *η*_p_^2^ = 0.063—while gender by age interaction was not significant. Univariate follow-up showed that boys reported significantly higher mean rating scores on Competence, Autonomy-choice, and Functional psychobiosocial states than girls. Moreover, 16–18-year-olds’ ratings were higher on Autonomy-choice but also on a Reduced sense of accomplishment and Sport devaluation than 13–15-year-olds’.

**Figure 1:**
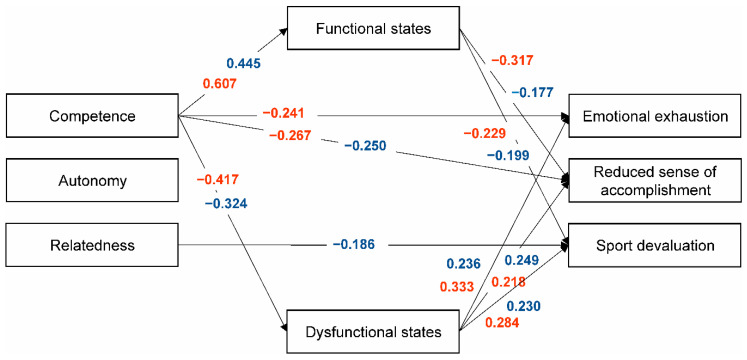
Path analysis results for the data of 13-15-year-olds. Only significant standardized estimates are presented (*p* < 0.05) for girls (red) and boys (blue).

**Figure 2:**
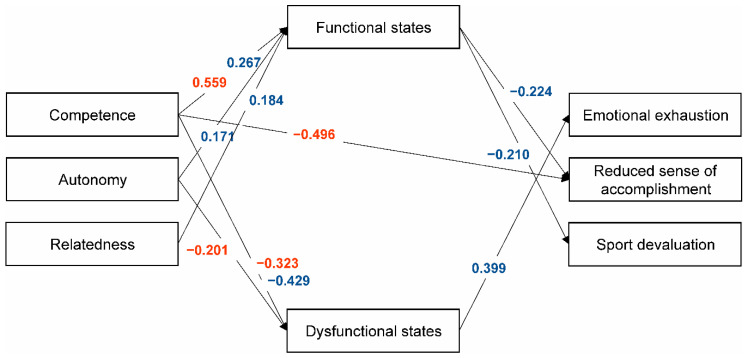
Path analysis results for the data of 16–18-year-olds. Only significant standardized estimates are presented (*p* < 0.05) for girls (red) and boys (blue).

Preliminary path analysis for the gender and age categories, in which all paths were estimated, indicated that not all the relationships between exogenous (i.e., independent) and endogenous (i.e., dependent) variables were significant. Models with significant standardized paths (see Figure 1 and Figure 2) yielded acceptable fit indices for all subgroups: 13–15-year-old girls, *χ*^2^(df) = 0.732 (2), CFI = 1.000, TLI = 1.000, RMSEA (90% CI) = 0.000 (0.000−0.137), and SRMR = 0.010; 13–15-year-old boys, *χ*^2^(df) = 7.236 (7), CFI = 0.999, TLI = 0.996, RMSEA (90% CI) = 0.015 (0.000−0.099), and SRMR = 0.046; 16–18-year-old girls, *χ*^2^(df) = 1.710 (1), CFI = 0.990, TLI = 0.910, RMSEA (90% CI) = 0.083 (0.000−0.290), and SRMR = 0.025; and 16–18-year-old boys, *χ*^2^(df) = 15.586 (9), CFI = 0.954, TLI = 0.908, RMSEA (90% CI) = 0.076 (0.000−0.138), and SRMR = 0.064.

**Figure 3:**
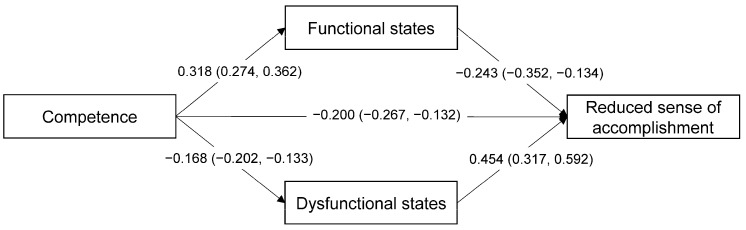
Parallel mediator model on the data of the whole sample. All non-standardized estimates (with 95% confidence intervals) are significant (*p* < 0.001).

Both the Competence and Functional/Dysfunctional psychobiosocial states were moderately correlated with a Reduced sense of accomplishment. Thus, further analysis was carried out to examine the possible mediating effects of Functional and Dysfunctional psychobiosocial states on the relationship between Competence and a Reduced sense of accomplishment using the Hayes’ [44] PROCESS computational tool. Mediation analysis was conducted on the data of the whole sample entering gender and age as covariates in a parallel multi-mediator model. The bias-corrected bootstrap confidence intervals for the regression coefficients were based on 5000 resamples. As represented in Figure 3, all the non-standardized paths were significant at *p* < 0.001, and the confidence intervals did not include the value zero. Thus, both direct and indirect effects of Competence on a Reduced sense of accomplishment were found. The variance in the Reduced sense of accomplishment accounted for by Competence was *R*^2^ = 0.243 and, together with psychobiosocial states, was *R*^2^ = 0.334. The completely standardized direct effect size was *ab_cs_* = −0.274, and the total effect size was *ab_cs_* = −0.484.

## 4. Discussion

The aim of this study was to examine the impact of psychological basic need satisfaction and psychobiosocial (i.e., functional and dysfunctional) states on burnout in young athletes. In recent years, there has been a growing interest in studying the role of basic psychological needs in the interplay between contextual characteristics and well-being (e.g., positive affect, enjoyment, and vitality) and ill-being (e.g., negative affect and emotional and physical exhaustion). Our study extends beyond research on basic need satisfaction and the related emotional responses by considering psychobiosocial states as mediators of burnout symptoms.

The findings indicate that the perception of the motivational environment was positive for our sample of young athletes (Table 2). Indeed, the scores on the basic need satisfaction scales and functional psychobiosocial states were relatively high, while the scores on the dysfunctional state and burnout scales were low. Nevertheless, it is worth noting that the scores in the Autonomy-choice subscale were lower than those in the Competence and Relatedness subscales. Although all of the three basic needs are regarded as essential for well-being and positive outcomes, autonomy, or the need to self-regulate one’s experiences and actions, is seen as the most critical aspect of supportive environments [25]. As expected, all the basic needs correlated positively with functional states and negatively with dysfunctional states and the three scales of burnout. Moreover, functional states correlated negatively with the scales of burnout, whereas dysfunctional states correlated positively with them. The moderate values of correlation between competence need satisfaction and functional states (positive correlation) and between competence and a reduced sense of accomplishment (negative correlation) proved competence to be a criterion of quality in youth sport settings and a component of meaningful experiences in sport participation [45].

Significant differences were found by age (13–15- vs. 16–18-year-olds), with older participants reporting higher scores on all the burnout scales (Emotional/physical exhaustion, Reduced sense of accomplishment, and Sport devaluation). Age differences were also shown in previous research on burnout. For example, in a three-year study on the trajectories of burnout development during adolescence, Ingrell, Johnson, and Ivarsson [46] observed increases in the scores for all the burnout variables. Harris and Watson [47] found 15–17-year-olds to report significantly more exhaustion and reduced sport accomplishment than younger children. These factors tend to increase during adolescence because of the augmented training loads associated with higher competitive levels. Higher levels of a reduced sense of sport accomplishment were also ascribed to a better capacity during growth to more realistically conceptualize the self’s sense of sport accomplishment derived from external available feedback [47].

In our study, we also observed significant differences by gender for the Competence, Autonomy, and Functional psychobiosocial states, with boys reporting higher scores. Competence perception is shaped through socialization processes and influenced by different socialization agents (e.g., parents, siblings, and coaches). Due to the gender characterization of activities, boys and girls go through experiences of success that are different and, therefore, contribute differently to the establishment of their sense of competence in a specific domain [48]. In both sport and physical domains, boys generally display greater competence and autonomy perception than girls [49,50,51,52]. According to Gill [51], gender differences reflect gender-role socialization, and, even in sport, the perceived gender differences are much greater than the actual differences. The characteristics and behaviors necessary for practicing sport disciplines, such as being strong, aggressive, and competitive, are stereotypically associated with masculinity and are in contrast with the stereotypical female characteristics. This represents a challenge to girls and women involved in sport and physical activity.

According to our hypotheses, path analysis showed the basic psychological need of Relatedness to be negatively associated with Sport devaluation in 13–15-year-old boys and positively associated with Functional states in 16–18-year-old boys (Figure 1 and Figure 2). Autonomy-choice was negatively related to Dysfunctional states in 16–18-year-old girls and positively related to Functional states in 16–18-year-old boys. Both direct and indirect negative relationships were found between Competence and the burnout variables across gender and age. The mediation analysis results by the age subsamples and of the whole sample showed Competence need satisfaction to be the most influential variable, with direct and indirect negative links to the burnout components, particularly a Reduced sense of sport accomplishment. Competence self-perceptions and beliefs are considered to be at the core of several motivational theories. Although conceptualized differently (e.g., self-concept, self-esteem, self-efficacy, and confidence), the sense of competence is considered as a universal aspect of being human and exerting a pervasive impact on daily life, cognition, and behavior [53]. In sport and physical domains, competence perceptions have been shown to be functionally associated with effort and performance [54,55,56]. In Self-Determination Theory, competence refers to the individuals’ basic need to feel able to operate effectively within their important life contexts, seeking out optimal stimulation and challenging activities [25]. Marsh and colleagues [53] suggest a possible subtle distinction between competence self-perceptions and competence need satisfaction. To perceive competence need satisfaction, people need to evaluate their performance in relation to some standards, such as through social comparisons with others in a specific context, externally established standards of excellence, and temporal comparisons of past performance in the same domain (e.g., a personal best [53]). These conditions typically characterize experiences in the sport context. Findings of the present study suggest that Competence need satisfaction directly counteracts the negative consequences of a Reduced sense of accomplishment and Emotional/physical exhaustion in younger participants. This is in line with the view that competence satisfaction is one of the most dominant factors related to burnout and dropout prevention (for reviews, see [57,58]).

The present study also provided evidence for the role of psychobiosocial states in burnout symptoms. Functional and dysfunctional psychobiosocial states were significant predictors in the following hypothesized directions: functional states were positively related to psychological need satisfaction and negatively so to burnout symptoms, while dysfunctional states showed the opposite trends (see Figure 1 and Figure 2). Previous studies grounded in BPNT have mainly considered need satisfaction as a potential contributor to positive motivational consequences. Our results support this assumption, indicating protective effects of functional psychobiosocial states regarding burnout. Ryan and Deci [25] have also argued that need satisfaction failure is manifested in diminished growth, integrity, and well-being. In the current study, dysfunctional psychobiosocial states were observed to mediate the relationship between competence and burnout, suggesting that too-difficult challenges, feelings of limited mastery, and negative feedback may trigger dysfunctional emotional states and enhance burnout symptoms. In a holistic approach to the study of emotions in sport, the IZOF model considers emotional experiences in a multidimensional manner and conceptualizes them as composite and multicomponent constructs reflecting person–environment relationships (for reviews, see [12,14]). The model incorporates several interactive modalities of psychobiosocial performance-related states encompassing (a) affective, cognitive, motivational, and volitional (psychological); (b) bodily-somatic and motor-behavioral (biological); and (c) operational and communicative (social) modalities. Noteworthily, burnout is also defined as a multifaceted phenomenon, a psychophysiological syndrome, and a dysfunctional condition associated with deleterious affective, cognitive, motivational, and behavioral consequences [59]. Therefore, preventing burnout in youth sport is a major issue that should be addressed using a developmental approach within an educational comprehensive context, in order to simultaneously influence the physical, emotional, and cognitive demands and consequences of sport participation [22,60]. As our findings suggest, coaches can create a functional motivational atmosphere in sport aimed at satisfying the basic psychological needs of youngsters, improving psychobiosocial states, and preventing burnout.

The practical implications derived from our study’s findings concur with those of previous investigations on basic psychological need satisfaction [21,22,23]. Extensive research in youth sport highlights the important role of coaches in providing young people with positive experiences to foster their motivation, sustain participation, and enhance the quality of sport engagement [3,60]. Within the framework of the Self-Determination Theory [25], the BPNT is considered to be one of the most relevant contemporary perspectives that can be used to understand and optimize the influence of coaches on the motivation of young athletes [61]. To promote the optimal sport experiences, motivation, and well-being of athletes, coaches need to fulfill athletes’ basic needs of autonomy, competence, and relatedness [62]. In a study with young handball players, Alesi et al. [63] showed that high levels of basic psychological need satisfaction were associated with a higher perception of a functional task-involving climate established by the coach and greater commitment in sport. Therefore, coaches play a vital role in promoting adaptive psychological outcomes in their athletes [64]. In 2003, Mageau and Vallerand [65] proposed a motivational model in which some coaches’ behaviors (defined as autonomy-supportive behaviors) exert a beneficial impact on athletes’ basic need satisfaction and, consequently, on intrinsic and self-determined motivation. An autonomy-supportive interpersonal style is characterized by behaviors aimed at: (a) providing choice within specific rules and limits; (b) providing a rationale for tasks and limits; (c) acknowledging the other person’s feelings; (d) allowing athletes opportunities to take initiatives; (e) providing non-controlling competence feedback; (f) avoiding overt control, guilt-inducing criticisms, and controlling statements; and (g) preventing ego involvement. It can be argued that these behaviors also have a positive impact on the other two basic psychological needs of competence and relatedness, as well as on functional psychobiosocial states. More recent studies are delving into these indications to develop a set of explicit need-supportive coaching behaviors [66]. For instance, coaches are encouraged to ask open questions to their athletes and support their initiatives concerning sport participation. Coaches should become more aware of their coaching practice and how they can play a key role in enhancing and maintaining athletes’ motivation and commitment.

Future research should address some of the limitations of the present study. In particular, we assessed psychobiosocial states using a global measure of functional and dysfunctional states [33] rather than using a discrete approach in which single psychobiosocial states are differentiated. Additional theoretical insights and applied indications could be derived from the study of single and interactive effects on burnout of the psychological (i.e., affective, cognitive, motivational, and volitional), biological (i.e., bodily-somatic and motor-behavioral), and social modalities (i.e., operational and communicative) that typify psychobiosocial states, and how these modalities relate to the satisfaction of basic needs. Another limitation is that our study design was correlational, and, although it was based on a priori theoretical considerations, firm assertions about the direction of causality cannot be drawn. Compared to cross-sectional and correlational study designs, longitudinal and experimental studies can facilitate a deeper understanding of the interplay among basic need satisfaction, psychobiosocial states, and burnout. Finally, beyond the age and gender differences found in this study, additional differences in basic psychological need satisfaction, psychobiosocial states, and burnout symptoms could be found by type of sport. Future research should investigate the psychological responses of participants involved in different sport disciplines, such as individual, team, combat, precision, endurance, choreographic, and adventure sports.

## 5. Conclusions

The purpose of the study was to investigate the relationship between the perceived satisfaction of basic psychological needs, psychobiosocial states, and burnout symptoms in young athletes. As expected, psychological need satisfaction, as a situational antecedent of burnout, was found to exert protective effects on burnout symptoms, while psychobiosocial states were found to mediate this relationship. In particular, functional states were positively linked to psychological need satisfaction and negatively related to burnout symptoms, while dysfunctional states were negatively linked to need satisfaction and positively associated with burnout symptoms. Among the psychological basic needs, competence need satisfaction was the most influential variable, with direct and indirect effects on burnout components.

In summary, the results suggest that functional psychobiosocial states, as consequences of environmental motivational aspects, can have a significant effect on contrasting burnout symptoms, while dysfunctional psychobiosocial states can increase the same symptoms. Overall, the findings support the usefulness of investigating psychobiosocial states as conceptualized within the IZOF framework to examine BPNT predictions in youth sport. The relationship among basic psychological need satisfaction, functional/dysfunctional psychobiosocial states, and burnout symptoms opens the way to new research perspectives that have both theoretical and practical implications.

## Figures and Tables

**Table 1:** Fit indices of the measures derived from confirmatory factor analysis.

Measures	*χ*^2^(df)	*χ*^2^/df	CFI	TLI	RMSEA (90% CI)	SRMR
BNSSS	250.858 (74)	3.390	0.916	0.896	0.069 (0.059−0.078)	0.055
BNSSS (two correlated errors)	166.069 (71)	2.339	0.955	0.942	0.051 (0.041−0.062)	0.049
PBS-ST	196.009 (89)	2.202	0.925	0.911	0.049 (0.039−0.058)	0.051
PBS-ST (two correlated errors)	166.975 (87)	1.919	0.944	0.932	0.043 (0.033−0.052)	0.047
ABQ	258.399 (87)	2.970	0.900	0.879	0.062 (0.054−0.071)	0.065
ABQ (two items removed)	178.669 (62)	2.882	0.926	0.907	0.061 (0.051−0.071)	0.050

*Note*: BNSSS = Basic Needs Satisfaction in Sport Scale (three-factor scale); PBS-ST = Psychobiosocial States Scale, Trait version; ABQ = Athlete Burnout Questionnaire; *χ*^2^(df) = chi-square (degrees of freedom); CFI = comparative fit index; TLI = Tucker–Lewis fit index; RMSEA = root mean square error of approximation; SRMR = standardized root mean square residual.

**Table 2:** Descriptive statistics, Pearson product-moment correlation coefficients, Cronbach’s alpha values (α), composite reliability (CR), omega coefficients (ω), and average variance extracted (AVE) for the latent variables for the whole sample (*N* = 507).

Measure	Latent Variable	Age 13–15 Years		Age 16–18 Years		1	2	3	4	5	6	7	α	CR	ω	AVE
		Girls (*n* = 116)	Boys (*n* = 160)	Girls (*n* = 104)	Boys (*n* = 127)											
		*M* ± *SD*	*M* ± *SD*	*M* ± *SD*	*M* ± *SD*											
BNSSS	1. Competence	4.90 ± 1.08	5.49 ± 0.85	4.98 ± 1.07	5.43 ± 0.84								0.833	0.829	0.835	0.489
	2. Autonomy-choice	3.88 ± 1.57	4.53 ± 1.28	4.40 ± 1.63	4.73 ± 1.25	0.35 *							0.826	0.814	0.831	0.525
	3. Relatedness	6.07 ± 1.18	6.10 ± 0.84	6.01 ± 0.96	6.07 ± 0.85	0.37 *	0.25 *						0.760	0.825	0.805	0.500
PBS-ST	4. Functional states	2.48 ± 0.61	2.73 ± 0.55	2.51 ± 0.56	2.69 ± 0.54	0.56 **	0.23 *	0.28 *					0.840	0.840	0.842	0.431
	5. Dysfunctional states	0.50 ± 0.41	0.47 ± 0.39	0.45 ± 0.35	0.56 ± 0.46	−0.36 *	−0.15	−0.17	−0.27 *				0.734	0.733	0.741	0.293
ABQ	6. Emotional/physical exhaustion	1.74 ± 0.64	1.78 ± 0.74	1.68 ± 0.70	1.78 ± 0.68	−0.14	−0.07	−0.08	−0.16	0.25 *			0.817	0.827	0.826	0.490
	7. Reduced sense of accomplishment	2.23 ± 0.74	2.03 ± 0.62	2.28 ± 0.74	2.37 ± 0.75	−0.46 **	−0.11	−0.22 *	−0.41 **	0.42 **	0.23 *		0.737	0.728	0.742	0.313
	8. Sport devaluation	1.37 ± 0.72	1.45 ± 0.73	1.68 ± 0.83	1.70 ± 0.93	−0.18	−0.09	−0.11	−0.25 *	0.28 *	0.31 *	0.44 **	0.806	0.813	0.813	0.594

*Note*. BNSSS = Basic Needs Satisfaction in Sport Scale (three-factor scale); PBS-ST = Psychobiosocial States Scale, Trait version; ABQ = Athlete Burnout Questionnaire. * Low correlations, ** moderate correlations.
